# Prospective association between organic food consumption and the risk of type 2 diabetes: findings from the NutriNet-Santé cohort study

**DOI:** 10.1186/s12966-020-01038-y

**Published:** 2020-11-09

**Authors:** Emmanuelle Kesse-Guyot, Pauline Rebouillat, Laurence Payrastre, Benjamin Allès, Léopold K. Fezeu, Nathalie Druesne-Pecollo, Bernard Srour, Wei Bao, Mathilde Touvier, Pilar Galan, Serge Hercberg, Denis Lairon, Julia Baudry

**Affiliations:** 1 Sorbonne Paris Nord University, Inserm, Inrae, Cnam, Nutritional Epidemiology Research Team (EREN), Epidemiology and Statistics Research Center – University of Paris (CRESS), F- 93017 Bobigny, France; 2 Toxalim (Research Center in Food Toxicology), University of Toulouse, INRA, ENVT, INP-Purpan, UPS, 31027 Toulouse, France; 3 Department of Epidemiology, College of Public Health, University of Iowa, Iowa City, IA 52246 USA; 4 Département de Santé Publique, Hôpital Avicenne, F-93017 Bobigny, France; 5 Aix Marseille Université, INSERM, INRA, C2VN, Marseille, France

**Keywords:** Organic food, Pesticides, Diabetes, Nutrition, Cohort

## Abstract

**Background:**

Organic food (OF) consumption has substantially increased in high income countries, mostly driven by environmental concerns and health beliefs. Lower exposure to synthetic pesticides has been systematically documented among consumers of organic products compared to non-consumers. While experimental studies suggest that pesticides currently used in food production may be associated with type 2 diabetes (T2D), no well-conducted prospective studies have investigated the potential association between consumption of organic products and the risk of T2D, controlling for potential confounding factors.

The objective of this prospective study was to estimate the association between OF consumption and the risk of T2D.

**Methods:**

A total of 33,256 participants (76% women, mean (SD) age: 53 years (14)) of the French NutriNet-Santé prospective cohort study who completed the organic food frequency questionnaire were included (2014–2019). The proportion of OF in the diet (as weight without drinking water) was computed. The associations between the proportion of OF in the diet (as 5% increment and as quintiles) and the risk of T2D were estimated using multivariable Hazard Ratio (HR) and 95% confidence interval (95% CI) derived from proportional hazards models adjusted for confounders (sociodemographic, anthropometric, lifestyle, medical and nutritional factors).

**Results:**

During follow-up (mean = 4.05 y, SD = 1.03 y, 134,990 person-years), 293 incident cases of T2D were identified. After adjustment for confounders including lifestyle (physical activity, smoking status, alcohol consumption) and nutritional quality of the diet assessed by the adherence to the French food-based dietary guidelines, OF consumption was associated with a lower risk of T2D. Participants with the highest quintile of OF consumption, compared with those with the lowest quintile, had 35% lower risk of T2D (95% CI = 0.43–0.97). Each increment of 5% in the proportion of OF in the diet was associated with 3% lower risk of T2D (HR 0.97, 95% CI = 0.95–0.99).

**Conclusions:**

In this large prospective cohort study, OF consumption was inversely associated with the risk of T2D. Further experimental and prospective studies should be conducted to confirm these observations.

**Clinical trial registry:**

The study was registered at ClinicalTrials.gov (NCT03335644).

**Supplementary Information:**

The online version contains supplementary material available at 10.1186/s12966-020-01038-y.

## Research in context

**What is already known about this subject? (maximum of 3 bullet points)**

- While plant-based organic foods, containing few pesticide residues, are perceived as healthier by consumers, there are few studies on the links between organic food consumption and health
- Experimental studies suggest a role of pesticides on diabetes development but epidemiological data are lacking

**What is the key question? (one bullet point only; formatted as a question)**

- Is organic food consumption associated with type 2 diabetes independently of confounders

**What are the new findings? (maximum of 3 bullet points)**

- In this population-based prospective cohort, after adjustment for sociodemographic, anthropometric, lifestyle, medical and dietary factors, an increase of 5 points in the proportion of OF in the diet was associated with a 3% decrease in T2D risk.
- Participants with the highest quintile of OF consumption, compared with those with the lowest quintile, had 35% lower risk of T2D (95% CI = 0.43–0.97).
- Upon confirmation, these findings suggest that potential benefits of organic food consumption may contribute to T2D prevention.

**How might this impact on clinical practice in the foreseeable future? (one bullet point only)**

- These findings warrant replication, confirmation, and investigation in particular to be able to draw causal inference in future studies.

## Introduction

The prevalence of type 2 diabetes (T2D) has significantly increased over recent decades, concomitantly to the obesity epidemic, making diabetes the fourth leading cause of global disability [1] and contributing largely to the global disability burden [2]. Currently in European countries, about 60 million people are living with diabetes and, in 2010 [3]. Diabetes is a multifactorial disease and scientific literature has underlined, beyond genetic and psychosocial factors, the crucial role of adiposity and several lifestyles-related factors, including physical activity, smoking, alcohol consumption and dietary patterns, in diabetes etiology [1].

According to a recent meta-analysis, there is a high level of evidence that processed meat, red meat and sweetened beverage consumption increases the risk of T2D, while whole grain consumption decreases the risk [4]. Other potential dietary factors involved in T2D etiology, although graded lower certainty, include fruit, vegetables, fish, nuts and dairy products (especially yoghurt) [4].

Among the emergent risk factors, exposure to environmental contaminants including pesticides is of major concern. Occupational exposure to pesticides has been associated with metabolic disorders [5–7]. In general population, exposure to pesticide residues is often multi-sources but diet is the most important route in the general population [8]. In particular, organochlorine compounds, highly persistent but now banned from agriculture in Europe, have been systematically associated with an increased risk of T2D [9]. Although scientific evidence for other types of pesticides is still limited, some epidemiological or experimental studies in animals support the biological plausibility that exposure to several classes of pesticides can affect the risk of obesity or diabetes [5, 10, 11]. Indeed, some specific molecules (pyrethroids, organophosphates, and organochlorides), are potential endocrine disruptors, responsible for possible metabolic disorders [12, 13]. For instance, a recent review reported a role of currently used organophosphorus, carbamate and pyrethroid pesticides in the incidence of diabetes [14]. In France, the organic agriculture is based on the European organic principles prohibiting use of synthetic fertilizers and pesticides and promoting the diversification of crops and livestock [15]. In terms of nutritional content, organic plant-based foods exhibit higher concentrations in some antioxidants such as carotenoids while organic animal-based foods exhibit higher concentrations in n-3 fatty acids [16]. Besides, a recent European Food Safety Authority report indicated that 44% of conventional crop-based food contained at least one quantifiable pesticide residue, vs 6.5% in organic-labeled foods (OF) [17]. In turn, OF consumption has been related to lower urinary pesticide exposure in a broad range of settings and different populations [16]. This is in line with our previous findings showing that high consumers of OF in subsample of the NutriNet-Santé cohort exhibited lower exposure to pesticide residues except for spinosad authorized in organic farming [18, 19]. Moreover, a recent cross-sectional study conducted in the United States found that participants in the National Health and Nutrition Examination Survey reporting purchasing OF had a 20% lower prevalence of diabetes c [20]ompared to those who did not purchase OF after adjustment for known diabetes-related risk factors .

In that context, the objective of the present study conducted in a large prospective cohort was 1) to investigate the prospective association between OF consumption and the risk of T2D, and 2) to estimate the mediation effect by the potential healthiness of a plant-based diet. Indeed, we hypothesize that OF consumers, as less exposed to pesticides residues from diet, may exhibit a lower risk of developing T2D. In addition, as we previously documented an association between OF preferences and a diet rich in plant-based food [18], we hypothesize that the association, if any, would be more pronounced among high plant-based food consumers, due to their higher intake of pesticide-contaminated foods.

## Material and methods

### Study population

The study is based on the data from the web-based prospective cohort NutriNet-Santé study conducted in France, initiated to investigate the relationship between determinants of dietary behaviors, nutrition and health. The rationale, design, and methodology have been described in detail previously [21]. Adult volunteers were enrolled from the general population and completed self-administered questionnaires using a dedicated platform about sociodemographic characteristics, lifestyle factors, health status, physical activity, anthropometric factors and diet.

The NutriNet-Santé study is conducted according to the Declaration of Helsinki guidelines and was approved by the Institutional Review Board of the French Institute for Health and Medical Research (IRB Inserm n°0000388FWA00005831) and the “Commission Nationale de l’Informatique et des Libertés” (CNIL n°908,450/n°909,216). The study protocol is recorded at Clinicaltrials.gov under the number: NCT03335644.

### Data collection

Food consumption was assessed through a previously validated self-administered semi-quantitative food frequency questionnaire (FFQ), including additional questions about frequencies consumption in the organic form (as based on certified and labeled products) for each item. The Organic FFQ has been described in details elsewhere [22]. In brief, the Organic FFQ included 264 items (food and beverages) with stated serving sizes or photographs. For each item, participants were asked to provide the frequency of consumption over the past year along with the quantities. The frequency consumption in the organic form was assessed for each item using the following question: ‘How often was the product of organic origin?’ with five modalities (never, rarely, half of time, often and always) and converted into quantitative data by assigning a weight to the consumption of the item of 0, 0.25, 0.5, 0.75 and 1 to the respective categories: ‘never’, ‘rarely’, ‘half the time’, ‘often’ and ‘always’. Consequently, the proportion of OF in the diet was calculated as the total OF consumption (in grams per day) divided by the total consumption without water (in grams per day). This ratio was also computed for plant- and animal- based food groups. Nutrient intakes were calculated using a food composition database (independent of the food production - organic or conventional), based on the NutriNet-Santé food composition table [23].

We also computed the sPNNS-GS2 (simplified *Programme National Nutrition San*té Guideline Score 2), a validated dietary quality score reflecting the adherence to the 2017 French dietary guidelines considering the principal guidelines, i.e. with no consideration for OF consumption [24]. Components, scoring and weighting are shown in S1 Table.

### Case ascertainment

Participants could report their health events using several methods: yearly health questionnaire, a specific health check-up questionnaire every six months, or at any time spontaneously through the platform. They were also prompted to declare all medication uses. Furthermore, due to a Decree in the Council of State (*n*°2013–175) that allows researchers to link data from independent cohorts to the medico-administrative databases of the National health insurance (SNIIRAM database), we were able to view participants medication and medical consultation history. T2D cases were therefore ascertained using a multi-source approach, i.e. T2D self-reported during follow-up along with declaration of the use of T2D medication (or a reimbursement of T2D medication detected from SNIIRAM).

### Covariates

At baseline, data on age, sex, highest graduation degree, occupation, income, smoking habits and physical activity were collected [25]. Income per household unit was calculated by dividing the household’s total monthly income by the number of consumption units (CU) [26]. The following categories of monthly income were used: < 1200; 1200-1800; 1800–2700 and > 2700 euros per household unit. Physical activity was assessed using the International Physical Activity Questionnaire (IPAQ) [27].Validated anthropometric questionnaires provided information on height and weight [28]. At baseline, antihypertensive and lipid-lowering medications as well as self-declaration of hypertension or dyslipidemia were declared through health questionnaire.

### Statistical analysis

**Fig. 1:**
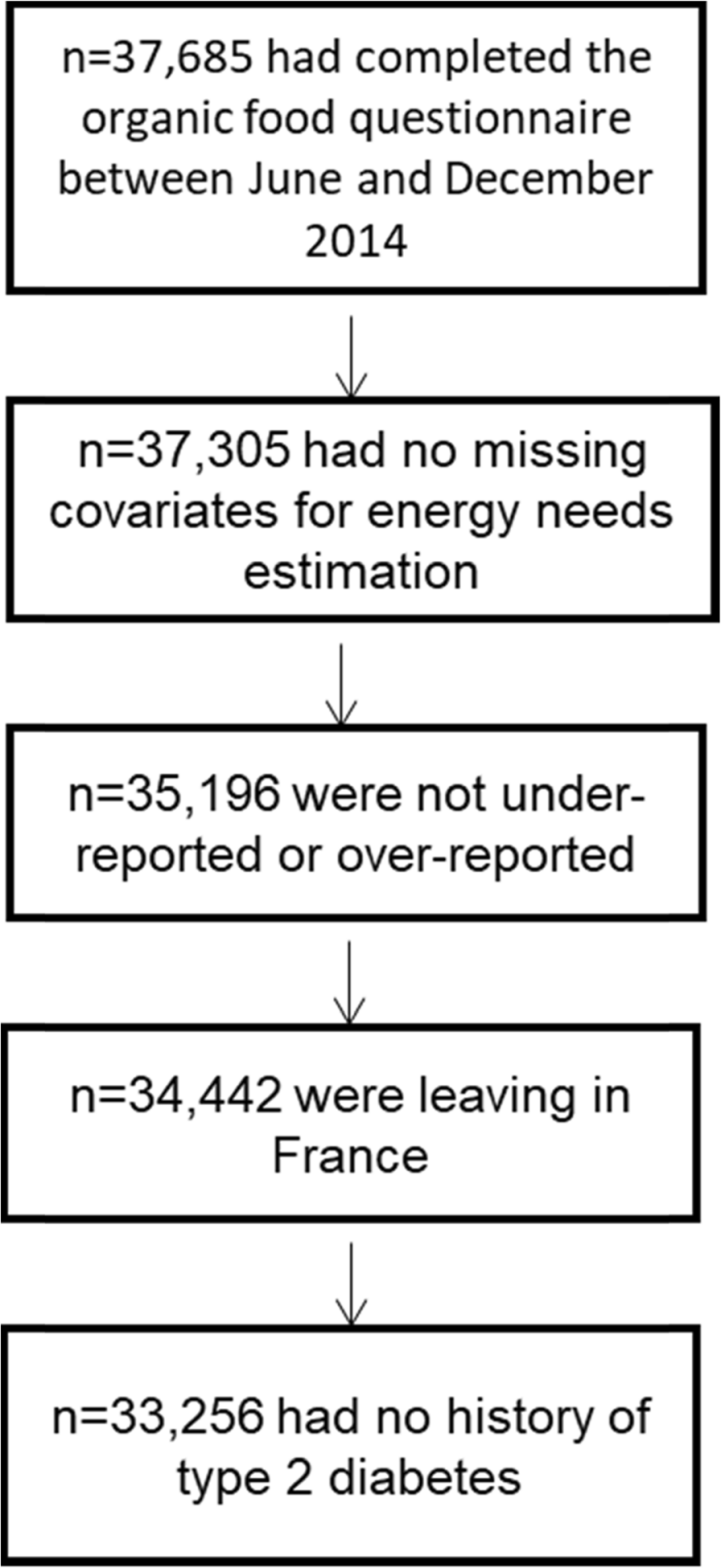
Selection of the sample

For this study, we retained NutriNet-Santé participants who completed the Organic FFQ between June and December 2014 (*N* = 37,685), with no missing data to compute energy needs as this was necessary to compute misreporting (*N* = 37,305), who were not detected as under- or over-reporters (*N* = 35,196), and who were living in France (*N* = 34,442). Finally, prevalent cases of T2D at baseline (*N* = 1186) were excluded, leading to an analysis sample of 33,256 participants (Fig. 1). Under- and over-reporters were those with a ratio between energy intake and energy requirement below or above cutoffs previously identified (0.35 and 1.93) and corresponding to the 1st and 99th percentile of the ratio distribution [22].

Baseline characteristics are presented by sex-specific quintile of the proportion of OF to the diet.

*P*-values referred to linear contrast across quintiles for continuous factors, Mantel-Haenzel Chi-square trend test for ordinal variables, and Chi-square test for categorical variables. Cox proportional hazards models using age as time-scale were used to estimate the relationship between OF consumption (modeled as a continuous variable (per 5% of increment of OF in the diet) and as sex-specific quintiles (the lowest quintile as reference)) and the risk of T2D providing hazard ratios (HR) and 95% confidence intervals (CI). Participants contributed person-time until the date of diabetes diagnosis, the date of last completed questionnaire or April, 19th 2019, whichever occurred first. Risk proportionality assumptions were estimated by examination of the Schoenfeld residuals. The first model was adjusted for gender, familial history of T2D, physical activity, occupation, marital status, education, monthly income per unit, smoking status, sPNNS-GS2, energy intake and alcohol consumption. The main model was further adjusted for BMI. Further adjustments for menopausal status, hormonal treatment (contraception or menopausal treatment) among women and living area (rural/urban) were tested and did not modify the results (data not shown).

We tested non-linear associations between OF consumption and T2D risk and verified the assumption of linearity using restricted cubic spline (RCS) functions with a SAS® macro [29]. We also stratified the analyses according to several factors which may potentially modify the associations, namely gender and high level of plant-based foods (according to tertiles of the sPNNS-GS2) and monthly income per household unit.

For comparability purpose with studies outside France, we have performed a sensitivity analysis by replacing the sPNNS-GS2 by the MEDI-LITE score [30]. As previously published, the MEDI-LITE, which ranges from 0 (least healthy) to 18 (healthiest), includes 9 components focusing on consumption of fruit, vegetables, whole grains, nuts and legumes, olive oil (positive points), dairy, red and processed meat (negative points), and alcohol (points according to consumption). Points are allocated according to a scoring system based on daily or weekly consumption.

To test the robustness of our findings, a set of sensitivity analyses encompassing removing of early cases or cases of cardiovascular diseases/metabolic disorders and additional adjustments (described in the supplemental material) were performed and we conducted a mediation analysis to estimate direct and indirect (through the nutritional quality of the diet) statistical effect of OF consumption on the risk of T2D.

All tests were two-sided, and *P* < 0.05 was considered statistically significant. SAS® version 9.4 (SAS Institute) and R software were used for the analyses.

## Results

The mean follow-up was 4.06 (SD = 1.03) years and the mean age at baseline was 52.95 (SD = 14.2) years old. During follow-up (134,990 person-years), 293 incident T2D cases were identified.

### Baseline characteristics of the sample

Characteristics of the participants are presented across quintiles of OF consumption (as proportion of OF in the whole diet) in Table 1. Higher OF consumption was positively associated with being older, more education, higher income or attaining a managerial position and to be physically active. Increase in OF in the diet was associated with higher energy intake and a lower contribution of proteins and higher contribution of carbohydrates and lipids to total daily energy intake. Higher consumption of OF was also associated with higher sPNNS-GS2 (reflecting adherence to dietary guidelines), lower alcohol consumption and lower body mass index.

**Table 1:** Baseline characteristics of the sample across sex-specific quintiles of the proportion of organic food in the diet, NutriNet-Santé study, 2014–2019, *N* = 33,256^*^

	Q1	Q2	Q3	Q4	Q5	P^1^
N	6650	6652	6652	6652	6650	
Cut-off of organic food Share						
Women	≤0.04	> 0.04–0.17	> 0.17–0.32	> 0.32–0.56	> 0.56	
Men	≤0.01	0.01–0.12	0.12–0.26	0.26–0.5	> 0.50	
Female (%)	76.33	76.32	76.32	76.32	76.33	–
Age (years)	52.7 (14.9)	52.5 (14.3)	53.3 (13.8)	53.8 (13.5)	53.7 (13.3)	< 0·0001
Education (%)						< 0·0001
< High school diploma	25.1	19.3	20.7	19.3	17.8	
High school diploma	16.6	15.3	13.8	13.6	13.9	
Post-secondary graduate	58.3	65.4	65.5	67.2	68.4	
Monthly income per unit household unit in € (%)						< 0·0001
Missing	6.74	5.97	6.21	6.06	6.32	
< 1200	14.81	10.96	10.37	9.86	11.94	
1200–1800	25.13	22.81	22.49	21.83	22.74	
1800–2700	25.5	27.6	27.27	28.16	27.53	
> 2700	27.82	32.67	33.66	34.1	31.47	
Occupational categories (%)						< 0·0001
Non-employed	4.41	3.76	3.97	3.85	4.87	
Retired	35.97	33.51	35.24	36.35	34.56	
Employee/Manual worker	19.23	16.07	14	11.91	12.6	
Intermediate profession	14.17	15.81	16.15	15.94	14.27	
Managerial staff	16.57	22.34	22.96	22.91	23.31	
Never employed	7.97	7.07	6.1	7.13	7.62	
Craftsman, shopkeeper, business owner, and farmer	1.68	1.44	1.59	1.92	2.77	
Cohabiting (%)	86.36	86.64	88.05	88.08	86.96	0.04
Tobacco status (%)						0.64
Former smoker	38.78	39.13	39.37	40.83	40.6	
Current smoker	12.8	11.68	11.79	9.89	9.2	
Never smoker	48.42	49.19	48.84	49.28	50.2	
Physical activity (%)						< 0·0001
Missing	23.16	21.2	20.11	19.35	16.19	
Low	25.08	22.34	19.69	17.61	15.29	
Medium	18.4	20.25	20.76	20.44	20.16	
High	17.81	17.99	19.31	21.11	23.77	
Energy intake (kcal/d)	1965 (648)	1980 (618)	1995 (611)	2022 (635)	2011 (618)	< 0·0001
% Carbohydrates	39.73 (7.72)	39.43 (7.38)	39.68 (7.37)	39.45 (7.27)	40.25 (7.82)	0.0003
% Lipids	40.29 (7.17)	40.77 (6.92)	40.87 (6.84)	41.59 (6.85)	42.38 (7.62)	0·0017
% Proteins	19.58 (3.82)	19.40 (3.49)	19.05 (3.50)	18.58 (3.52)	17.00 (3.51)	< 0·0001
Alcohol consumption (g/d)	8.54 (14.31)	8.65 (12.41)	8.62 (11.91)	8.58 (11.73)	7.41 (10.53)	< 0·0001
sPNNS-GS2	1.83 (3.48)	2.16 (3.40)	2.60 (3.30)	2.99 (3.29)	4.29 (3.03)	< 0·0001
Body mass index (kg/m^2^)	24.77 (4.86)	24.25 (4.65)	24.11 (4.36)	23.84 (4.24)	22.97 (3.89)	< 0·0001
Family history of T2D	18.45	17.51	16.58	16.37	16.75	0.002
History of hypertension	17.98	16.27	15.53	14.51	10.75	< 0·0001
History of dyslipidemia	19.85	18.72	18.64	18.13	13.31	< 0·0001

*Abbreviations*: *Q* sex-specific Quintile

^*^All variables were assessed at baseline

^1^P for linear contrast or Chi^2^ test

### Proportion of OF in the diet and T2D

**Fig. 2:**
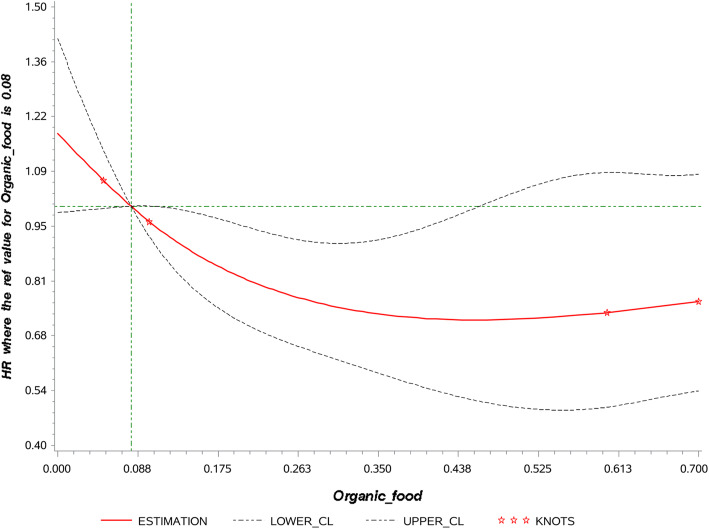
Linearity assumption of the association between the proportion of organic food in the diet and type 2 diabetes risk^1,2^. ^1^Spline plot modeling the association between the proportion of organic food in the diet and type 2 diabetes risk using Restricted cubic spline (RCS) SAS Macro® developed by Desquilbet and Mariotti. ^2^ *P* for overall association = 0.02, *P* for non-linear association = 0.12

The association between OF consumption (as proportion in the diet) and risk of T2D is presented in Table 2. The linearity of the association is illustrated in Fig. 2. No evidence of non-linearity was observed. After adjustment for confounders including lifestyle, sociodemographic data and quality of the diet, higher OF consumption was inversely associated with T2D risk (HRQ5 vs. Q1 = 0.64, 95%CI = 0.43–0.95, *P* for trend = 0.01). Each increment of 5% in the proportion of OF in the diet was associated with 3% reduction in risk of T2D (HR 0.97, 95% CI = 0.94–0.99). After further adjustment for BMI, relationships were marginally attenuated (HRQ5 vs. Q1 = 0.65, 95%CI = 0.43–0.97, P for trend = 0.01; HR per 5% increment = 0.97 95%CI = 0.95–0.99). When considering the proportion of OF in the consumption of plant products, findings were similar although slightly attenuated (HR 5% increment = 0.98, 95%CI = 0.95–1.00, *P* = 0.03). Of note, the point estimate for Q5 was higher than for Q4. For animal food products, the association was not statistically significant.

**Table 2:** Association between the proportion of organic food in the diet and risk of T2D, NutriNet-Santé study, 2014–2019, *N* = 33,256^*^

model	Q1	Q2	Q3	Q4	Q5	P for trend^**1**^	5 points increment	P^**2**^
***Total organic food***								
Person-years	26,709	27,128	27,218	27,120	26,816			
Number of cases	83	72	51	48	39			
Model 1^3^	1.00 (ref)	0.94 (0.69, 1.30)	0.69 (0.48, 0.98)	0.70 (0.48, 1.00)	0.64 (0.43, 0.95)	0.01	0.97 (0.94, 0.99)	0.01
Model 2^4^	1.00 (ref)	0.89 (0.65, 1.23)	0.69 (0.48, 0.98)	0.67 (0.46, 0.96)	0.65 (0.43, 0.97)	0.01	0.97 (0.95, 0.99)	0.02
***Organic plant food***								
Person-years	26,733	27,141	27,179	27,239	26,698			
Number of cases	83	67	55	40	48			
Model 3^5^	1.00 (ref)	0.88 (0.63, 1.21)	0.74 (0.53, 1.05)	0.55 (0.38, 0.81)	0.76 (0.52, 1.10)	0.01	0.97 (0.95, 1.00)	0.03
Model 4^6^	1.00 (ref)	0.83 (0.60, 1.16)	0.75 (0.53, 1.06)	0.52 (0.36, 0.77)	0.77 (0.53, 1.12)	0.01	0.98 (0.95, 1.00)	0.03
***Organic animal food***								
Person-years	26,541	27,104	27,236	27,148	26,960			
Number of cases	75	58	65	46	49			
Model 5^7^	1.00 (ref)	0.81 (0.57, 1.14)	0.93 (0.66, 1.30)	0.68 (0.47, 0.99)	0.84 (0.58, 1.22)	0.19	0.99 (0.96, 1.01)	0.27
Model 6^8^	1.00 (ref)	0.77 (0.54, 1.09)	0.94 (0.67, 1.31)	0.64 (0.44, 0.93)	0.82 (0.56, 1.19)	0.15	0.99 (0.96, 1.01)	0.27

*Abbreviation*: *Q* sex-specific quintile

^*^Values are Hazard ratio (95% confidence intervals), 5 points correspond to 5% of the contribution of OF to the diet

^1^P for trend modeling quintile as ordinal independent variable

^2^P for continuous independent variable

^3^Model 1 is adjusted for age (time-scale), gender, familial history of diabetes, physical activity, occupation, marital status, education, monthly income per unit, smoking status, sPNNS-GS2, energy intake and alcohol consumption

^4^Model 2 is model 1 adjusted for body mass index

^5^Model 3 is model 1 adjusted for total plant food consumption

^6^Model 4 is model 3 adjusted for body mass index

^7^Model 5 is model 1 adjusted for total animal food consumption

^8^Model 6 is model 5 adjusted for body mass index

### Stratified analysis

Stratified analyses are shown in Table 3**.** The inverse association between OF consumption and T2D risk was more specifically observed in women and in participants with a high level of adherence to the food-based guidelines. The associations were similar but statistically significant (statistical power was reduced) only among low and high-income individuals. No significant associations were observed in other strata.

**Table 3:** Stratified analysis of the association of the proportion of organic food in the diet and risk of T2D, NutriNet-Santé study, 2014–2019^*^

	Q1	Q2	Q3	Q4	Q5	P for trend^**1**^	5 points increment	P^**2**^	P for interaction
**Sex**									
Women^3^	1.00 (ref)	0.66 (0.43, 1.00)	0.67 (0.43, 1.03)	0.51 (0.31, 0.83)	0.35 (0.19, 0.63)	0.0002	0.94 (0.91, 0.97)	0.001	0.01
Men^4^	1.00 (ref)	1.38 (0.81, 2.33)	0.75 (0.40, 1.41)	1.22 (0.68, 2.17)	1.61 (0.89, 2.91)	0.28	1.03 (0.99, 1.07)	0.15	
**sPNNS-GS2**									
Tertile 1	1.00 (ref)	0.75 (0.47, 1.21)	0.78 (0.48, 1.26)	0.55 (0.32, 0.93)	0.72 (0.43, 1.20)	0.08	0.99 (0.95, 1.03)	0.59	0.24
Tertile 2	1.00 (ref)	1.17 (0.65, 2.12)	0.66 (0.33, 1.30)	0.70 (0.36, 1.39)	0.55 (0.26, 1.15)	0.04	0.96 (0.91, 1.00)	0.07	
Tertile 3	1.00 (ref)	0.60 (0.28, 1.28)	0.56 (0.26, 1.21)	0.48 (0.20, 1.11)	0.42 (0.17, 1.03)	0.04	0.95 (0.90, 1.00)	0.06	
**Monthly income per unit household unit in €**									
< 1200^5^	1.00 (ref)	0.69 (0.30, 1.56)	0.36 (0.13, 0.97)	0.53 (0.20, 1.44)	0.20 (0.04, 0.94)	0.02	0.92 (0.84, 1.00)	0.05	0.99
1200–1800^6^	1.00 (ref)	0.58 (0.28, 1.22)	0.69 (0.33, 1.42)	0.78 (0.38, 1.62)	0.55 (0.22, 1.35)	0.32	0.97 (0.91, 1.02)	0.25	
1800–2700^7^	1.00 (ref)	1.09 (0.53, 2.21)	0.95 (0.46, 1.98)	1.27 (0.62, 2.60)	1.04 (0.46, 2.39)	0.75	1.01 (0.97, 1.07)	0.56	
> 2700^8^	1.00 (ref)	0.85 (0.49, 1.45)	0.50 (0.26, 0.96)	0.38 (0.19, 0.79)	0.65 (0.33, 1.25)	0.02	0.95 (0.90, 0.99)	0.03	

*Abbreviations*: *CI* confidence interval, *HR* Hazard ratio, *PNNS-GS2* Programme National Nutrition Santé-guideline score 2, *Q* sex-specific (when appropriate) quintile, *T2D* type 2 diabetes

^*^Values are Hazard ratio (95% confidence intervals). 5 points correspond to 5% of the contribution of OF to the diet. Model are adjusted for age (time-scale), familial history of diabetes, physical activity, occupation, marital status, education, monthly income per unit, smoking status, sPNNS-GS2, energy intake, alcohol consumption and body mass index and gender for sPNNS-GS2 stratification

^1^P for trend modeling quintile as ordinal independent variable

^2^P for continuous independent variable

^3^*N* = 25,383

^4^*N* = 7873

^5^*N* = 3854

^6^*N* = 7648

^7^*N* = 9050

^8^*N* = 10,623

### Sensitivity analysis

Excluding early (during the first 2 years of follow-up) cases of T2D (to limit reverse causality) or participants developing cardiovascular diseases during the follow-up led to similar findings. However, excluding participants with hypertension or dyslipidemia (final sample *N* = 24,316) at baseline or during the follow-up strongly attenuated the association (S2 Table).

Accounting for additional dietary or nutritional factors (described in the Supplemental Material) potentially related to T2D risk and OF consumption did not modify the findings (S2 Table).

Using the residues of OF consumption on dietary quality as main exposure substantially attenuated the findings although the association remained (HR per 5% increment = 0.98, 95%CI = 0.96, 1.00; *P* = 0.04) (S2 Table).

Finally, the mediation analysis aiming to better explain the causal chain is presented in S3 Table. The indirect effect of OF consumption through the quality of the diet was small among participants with low OF consumption. However, among those in the highest quintile of the proportion of OF in the diet, the association between OF consumption and the risk of T2D was partially mediated (up to 32%) by a healthy diet and the direct effect was estimated to a reduction in T2D risk of 28%.

Replacing sPNNS-GS2 by adherence to Mediterranean diet (using MEDI-LITE score) provided similar findings (S4 Table).

## Discussion

In the present study conducted in a large sample from the French adult population, an inverse association between OF consumption and T2D risk was observed. This association persisted after accounting for potential confounding effects of various factors, including lifestyles, dietary patterns and adiposity. Of note, the inverse relationship was observed more specifically among women and high consumers of plant-based foods.

Epidemiological evidence dealing with potential links between OF consumption and metabolic-related outcomes are scarce [20, 31, 32]. Two performed in the NutriNet-Santé study have shown that a high level of OF consumption is associated with a significantly lower probability of overweight and obesity in both men and women [31] as well as a reduced risk of metabolic syndrome [32]. An inverse association between BMI and OF consumption has been previously observed in this cohort thus adjustment for BMI has been performed in the models.

To the best of our knowledge, the present study is the first prospective cohort study to examine the association between OF consumption and T2D risk. Consistent with our findings, a recent cross-sectional study, found that frequent OF purchase, as a proxy of higher consumption of OF was inversely associated with diabetes prevalence among adults in the United States [20].

As healthy diets have been positively correlated with OF consumption in our cohort and other studies [18, 33, 34], it can be assumed that our findings can be, at least partially, explained by healthier diet of high consumers of OF. However, adjustment for dietary factors (specific and holistic) only marginally attenuated the association. Similarly, the mediation analysis carried out to quantify the direct and indirect effects of OF consumption on the risk of T2D suggested a specific role of OF consumption beyond the healthiness of the diet of high OF consumers.

With regard to type of organic foods, organic plant-food consumption was specifically associated with a reduced T2D risk in our study while no clear-cut association was observed with organic animal-food consumption. We observed a lower point estimate in Q5 than in Q4. It cannot be ruled that this association is an artefact resulting from an underlying association rather than a true association or due to imprecise estimation or reverse causation. Further studies investigating specific associations on detailed food groups may help to better understand this finding.

Furthermore, in the stratified analyses leading to a lower statistical power, a lower risk of T2D was only detected in participants exhibiting high level of adherence to food-based dietary guidelines (correlated to plant-based food consumption).

It is also noteworthy that we did not observe an inverse association among men.

In fact, a higher risk was observed among men who were OF consumers, although not statistically significant, while a strong link was detected among women. In the NHANES analysis [20], inverse association between OF and diabetes was similar among men and women (OR < 1) but did not reach statistical significance due possibly to limited statistical power.

The sex specific association may be biased by the lack of statistical power among men (*N* = 120 cases only). However, a sexual dimorphic response to pesticide exposure has been already reported in population-based [35] and in animal experiment [36–38] studies. Moreover, the sexual dimorphism in response to pesticide exposure may be related to different detoxifying capacities between males and females, or difference in regulation of the microbiota [39]. Further research is needed to clarify the sex-specific association of OF consumption with T2D risk.

The underlying mechanisms of the observed relationships can be seen in light of the differences in bioactive component contents between organic and conventional foods. First, some literature reviews have documented higher concentrations of some antioxidant compounds in organic plant-food [16]. As to carotenoids, this has also been established in a subsample of the NutriNet-Santé study comparing plasma concentrations of matched low and high OF consumers with similar characteristics including sociodemographic data, lifestyles and profiles of dietary consumption [40]. These antioxidants may play a marginal role in the etiology of T2D by reducing oxidative stress that disrupts glucose absorption by cells and can improve insulin sensitivity [41, 42].

Second, fatty acids profiles are potentially different in organic and conventional animal foods [16]. Organic meat and milk exhibit higher contents in n-3 fatty acids which may play a role against insulin-resistance. Differential fatty acids profiles have also been observed in low vs high OF consumers [40]. However, in the present study, a higher organic animal-based food consumption was not associated with lower T2D risk (in the whole sample) whereas this was observed in the American study [20]. This may be due to differences in food composition, levels of food consumption or differences in nutritional profiles between the two populations studied.

The third hypothesis relies on the fact that organic plant foods contain much less synthetic pesticide residues than their conventional counterparts [17] due to specifications and regulations prohibiting the use of these molecules during production, storage and processing. This is translated into different human exposures as checked by experimental and observational epidemiological studies [19, 43–47], documenting lower urinary concentration in some pesticide residues in relation to OF consumption. Scientific studies acknowledge that most pesticide moieties act as endocrine disruptors [48]. Harmful effects of organochlorine pesticides, which are now widely prohibited, on the development of T2D have been well documented [9]. Although less often studied, currently used pesticides such as organophosphorus, pyrethroids and neonicotinoids may also display properties involved in the etiology of T2D [5]. Experimental studies have reported an effect of low doses of these molecules on various mechanisms involved in the dysregulation of glucose hemostasis and insulin resistance including inflammatory pathways, disturbance of oxidative homeostasis, mitochondrial dysfunction, endocrine disruption and epigenetic alterations [11, 13, 49]. In our study, a stronger negative association between OF consumption and risk of T2D was observed in individuals with higher intakes of plant-based foods (i.e. who complied better with dietary recommendations). Indeed, the subgroup of individuals with the highest consumption of plant foods were those who benefited most from consuming OF in terms of T2D risk. Thus, the noticeably reduced pesticides residue exposure provided by organic plant foods is consistent with mechanistic data and support this hypothesis.

These findings are of public health relevance. Indeed, if our results are confirmed by research in other settings and populations and coupled with specific experimental studies, organic food consumption promotion may serve in T2D prevention strategies, as part of a dimension of a healthy diet.

Some limitations of our study should be noted. First, as the design is observational, causal inference is limited and residual confounding cannot be entirely ruled out. However, a wide range of sensitivity analyses were conducted to test the robustness of our findings. Second, generalizability to other populations is limited due to the characteristic of this population composed of volunteers, as illustrated by the low incidence of T2D which may have lowered the statistical power. Indeed, the participants included in this cohort were more often women, younger, with higher formal education, healthier dietary patterns and higher consumption of OF [22, 50, 51] compared to the general French population. However, the latter allows estimating the role of OF consumption at higher levels than those generally observed population. Third, the ascertainment of T2D cases was based on several sources but non-identification of some cases of diabetes cannot be totally excluded. Next, this study is based on self-reported data, in particular dietary consumption, which are prone to measurement errors and desirability bias. However, our previous work showed that organic and non-organic consumers exhibited differences in concentrations of some urinary pesticide residues which provides some evidence for the reliability of our nutritional data [19]. Finally, the follow-up of 4 years was relatively short, so that there may be reverse causality (i.e., people with subclinical diabetes chose to eat more organic foods instead of non-organic foods).

The strengths of our study include large sample size with various dietary patterns, in particular high OF consumers allowing assessment of the potential role of high proportion of OF in the diet with sufficient statistical power. In addition, for the first time, the OF consumption was assessed quantitatively for a high number of food items allowing an accurate estimation of the overall proportion of OF in the diet. Finally, the prospective design improved causal inference.

## Conclusion

In conclusion, this prospective study supports an inverse association between OF consumption, in particular organic plant-based foods, and the risk of T2D, in adults, especially women. Randomized trials would be helpful to fully demonstrate causal inference but such long-term interventional studies raise concerns about logistical feasibility. Overall, further investigations in observational studies in other settings and carefully designed randomized controlled trials are required to replicate these findings for confirmation purpose and to elucidate underlying mechanisms.

## Supplementary Information

**Additional file 1 Table S1.** Components, scoring and weighting used for sPNNS-GS2 cFigureS4ek. **Table S2**. Sensitivity analyses of the associations between organic food consumption and risk of T2D, NutriNet-Santé study, 2014–2019*. Abbreviations: CI: confidence interval, HR: Hazard ratio, PNNS-GS2: Programme National Nutrition Santé-guideline score 2, Q: quintile, T2D: type 2 diabetes. ^*^ Values are mean differences (95% confidence intervals). 5 points correspond to 5% of the contribution of OF to the diet. Model are adjusted for age (time-scale), gender, familial history of diabetes, physical activity, occupation, marital status, education, monthly income per unit, smoking status, sPNNS-GS2, energy intake, alcohol consumption and body mass index. ^**1**^P for continuous independent variable. ^2^T2D cases occurring during the first year of follow-up were excluded from the sample (final *n* = 33,162. 199 cases). ^3^Participants developing a cardiovascular disease during the follow-up or before were excluded from the sample (final *n* = 32,468, 273 cases). ^4^Participants with hypertension or dyslipidemia at baseline were removed (final *n* = 24,316. 85 cases). ^5^Model was further adjusted for consumption of processed meat, whole grain and sweetened beverages. ^6^Model was further adjusted for intake of saturated fatty acids, sugar, sodium and fiber. ^7^Residual of the regression of organic food consumption on sPNNS-GS2 was considered as principal exposure. ^8^Model with sPNNE-GS2 replaced by 4 dietary factors extracted by principal component analysis. **Table S3.** Mediation of the association between organic food consumption and risk of T2D by a healthy diet, NutriNet-Santé study, 2014–2019, *N* = 33,256*. Abbreviations: Q, Quintile; HR, hazard ratio. ^*^ The direct effect corresponds to the direct relation between organic food consumption and T2D, and the indirect effect corresponds to the relation mediated by sPNNS-GS2 (diet quality, in deciles) as described in the supplemental material. Values are hazard ratio and 95% confidence interval adjusted for age (time-scale), gender, family history of diabetes, physical activity, occupation, marital status, education, monthly income per unit, smoking status, energy intake, alcohol consumption and body mass index. ^1^Values correspond to the percentage of the total association mediated by sPNNS-GS2. **Table S4.** Association between the proportion of organic food in the diet and risk of T2D, adjusted for the Medi-Lite score, NutriNet-Santé study, 2014–2019, *N* = 33,256^*^. Abbreviation: Q: sex-specific quintile. ^*^Values are Hazard ratio (95% confidence intervals), 5 points correspond to 5% of the contribution of OF to the diet. ^**1**^P for trend modeling quintile as ordinal independent variable. ^2^P for continuous independent variable. ^3^Model 1 is adjusted for age (time-scale), gender, familial history of diabetes, physical activity, occupation, marital status, education, monthly income per unit, smoking status, MEDI-LITE, energy intake and alcohol consumption. ^4^Model 2 is model 1 adjusted for body mass index. ^5^Model 3 is model 1 adjusted for total plant food consumption. ^6^Model 4 is model 3 adjusted for body mass index. ^7^Model 5 is model 1 adjusted for total animal food consumption. ^8^Model 6 is model 5 adjusted for body mass index. **S5 Material.**

## Data Availability

The datasets generated and/or analyzed during the current study are not publicly available due protection under the protection of health data regulation set by the French National Commission for Information Technology and Liberties (Commission Nationale de l’Informatique et des Libertés, CNIL). The data are available upon reasonable request to the study’s operational manager, Nathalie Pecollo (dev@null), for review by the steering committee of the NutriNet-Santé study.

## Abbreviations

BMI: Body mass index
CU: Consumption units
HR: Hazard ratio
OF: Organic food
PNNS-GS2: Programme National Nutrition Santé-Guideline Score 2
PNNS-GS 2: Programme National Nutrition Santé-Guideline Score
Q: Quintile
T2D: Type 2 diabetes
