# Supplementary material for: Prospective association between organic food consumption and the risk of type 2 diabetes: findings from the NutriNet-Santé cohort study
Source: Int J Behav Nutr Phys Act. 2020 Nov 9;17:136. doi: 10.1186/s12966-020-01038-y (PMC7653706; doi:10.1186/s12966-020-01038-y)
Supplement: Supplementary file 1 — Additional file 1 Table S1. Components, scoring and weighting used for sPNNS-GS2 cFigureS4ek. Table S2. Sensitivity analyses of the associations between organic food consumption and risk of T2D, NutriNet-Santé study, 2014–2019*. Abbreviations: CI: confidence interval, HR: Hazard ratio, PNNS-GS2: Programme National Nutrition Santé-guideline score 2, Q: quintile, T2D: type 2 diabetes. * Values are mean differences (95% confidence intervals). 5 points correspond to 5% of the contribution of OF to the diet. Model are adjusted for age (time-scale), gender, familial history of diabetes, physical activity, occupation, marital status, education, monthly income per unit, smoking status, sPNNS-GS2, energy intake, alcohol consumption and body mass index. 1P for continuous independent variable. 2T2D cases occurring during the first year of follow-up were excluded from the sample (final n = 33,162. 199 cases). 3Participants developing a cardiovascular disease during the follow-up or before were excluded from the sample (final n = 32,468, 273 cases). 4Participants with hypertension or dyslipidemia at baseline were removed (final n = 24,316. 85 cases). 5Model was further adjusted for consumption of processed meat, whole grain and sweetened beverages. 6Model was further adjusted for intake of saturated fatty acids, sugar, sodium and fiber. 7Residual of the regression of organic food consumption on sPNNS-GS2 was considered as principal exposure. 8Model with sPNNE-GS2 replaced by 4 dietary factors extracted by principal component analysis. Table S3. Mediation of the association between organic food consumption and risk of T2D by a healthy diet, NutriNet-Santé study, 2014–2019, N = 33,256*. Abbreviations: Q, Quintile; HR, hazard ratio. * The direct effect corresponds to the direct relation between organic food consumption and T2D, and the indirect effect corresponds to the relation mediated by sPNNS-GS2 (diet quality, in deciles) as described in the supplemental material. Values are h [file 12966_2020_1038_MOESM1_ESM.docx]

**S1 Table: Components, scoring and weighting used for sPNNS-GS2 computation**

| **Dietary components** | **Recommendation** | **Criteria** | **Score** |
| --- | --- | --- | --- |
| Fruit and | At least 5 serv/d, | [0 - 3.5[ | 0 |
| vegetables | with 1 max as juice and 1 | [3.5 - 5[ | 0.5 |
| (weight=3) | max as dried | [5 - 7.5[ | 1 |
|  |  | ≥7.5 | 2 |
| Nuts | A handful/d | 0 | 0 |
| (weight=1) |  | )0 – 0.5[ | 0.5 |
|  |  | [0.5- 1.5[ | 1 |
|  |  | ≥1.5 | 0 |
| Legumes | At least 2 serv/w | 0 /w | 0 |
| (weight=1) |  | )0-2[ /w | 0.5 |
|  |  | ≥2 /w | 1 |
| Whole-grain | Every day | 0 | 0 |
| Food |  | )0 - 1[ | 0.5 |
| (weight=2) |  | [1 - 2[ | 1 |
|  |  | ≥2 | 1.5 |
| Milk and | 2 serv/d | [0 - 0.5[ | 0 |
| dairy products |  | [0.5 - 1.5[ | 0.5 |
| (weight=1) |  | [1.5 - 2.5[ | 1 |
|  |  | ≥2.5 | 0 |
| Red meat | Limit consumption | ≥750 g/w | -2 |
| (weight=2) |  | [500 - 750[ g/w | -1 |
|  |  | [0 - 500[ g/w | 0 |
| Processed meat | Limit consumption | ≥300 g/w | -2 |
| (weight=3) |  | [150 - 300[ g/w | -1 |
|  |  | [0 - 150[ g/w | 0 |
| Fish and | 2 serv/w | [0 - 1.5[serv /w | 0 |
| Seafood |  | [1.5 - 2.5[serv /w | 1 |
| (weight=2) |  | [2.5 - 3.5[serv /w | 0.5 |
|  |  | ≥3.5 serv /w | 0 |
| Added fat | Avoid overeating | >16% of EIWA ^c^ | 0 |
| (weight=2) |  | ≤16% of EIWA | 1.5 |
| Sugary foods | Limit consumption | ≥15% of EIWA | -2 |
| (weight=3) |  | [10-15[% of EIWA | -1 |
|  |  | <10 % of EIWA | 0 |
| Sweet-tasting | Limit consumption | ≥ 750mL mL/d | -2 |
| beverages |  | [250 - 750[ mL/d | -1 |
| (weight=3) |  | )0 - 250[ mL/d | -0.5 |
|  |  | 0 mL/d | 0 |
| Alcoholic | Limit consumption | >200 g/d | -2 |
| beverages |  | )100-200) g/d | -1 |
| (weight=3) |  | )0-100) g/d | 0 |
|  |  | 0 g/d | 0.5 |
| Salt | Limit consumption | >12 g/d | -2 |
| (weight=3) |  | )10-12) g/d | -1 |
|  |  | )8-10) g/d | -0.5 |
|  |  | )6-8) g/d | 0 |
|  |  | ≤6 g/d | 1 |

Abbreviations: d: day; EIWA: Energy intake without alcohol; w: week;

**S2 Table: Sensitivity analyses of the associations between organic food consumption and risk** **of T2D, NutriNet-Santé study, 2014-2019**^*^

|  | **Q1** | **Q2** | **Q3** | **Q4** | **Q5** | **P for trend** | **5 point increment**  **HR (95% CI)** | **P**^†^ |
| --- | --- | --- | --- | --- | --- | --- | --- | --- |
| Removing early cases^‡^ | 1.00 (ref) | 0.93 (0.64, 1.35) | 0.53 (0.33, 0.83) | 0.71 (0.46, 1.08) | 0.49 (0.29, 0.81) | 0.002 | 0.95 (0.92, 0.98) | 0.002 |
| Removing CVD^§^ | 1.00 (ref) | 0.83 (0.60, 1.16) | 0.64 (0.44, 0.92) | 0.62 (0.42, 0.90) | 0.60 (0.40, 0.91) | 0.002 | 0.97 (0.94, 0.99) | 0.01 |
| Removing metabolic abnormalities^\|\|^ | 1.00 (ref) | 0.81 (0.44, 1.47) | 0.69 (0.37, 1.31) | 0.55 (0.27, 1.11) | 0.52 (0.24, 1.12) | 0.04 | 0.96 (0.91, 1.00) | 0.07 |
| Food groups^¶^ | 1.00 (ref) | 0.89 (0.65, 1.23) | 0.68 (0.48, 0.98) | 0.66 (0.46, 0.95) | 0.63 (0.42, 0.95) | 0.005 | 0.97 (0.94, 0.99) | 0.02 |
| Nutrient intakes^#^ | 1.00 (ref) | 0.88 (0.64, 1.21) | 0.67 (0.47, 0.96) | 0.66 (0.46, 0.95) | 0.62 (0.41, 0.93) | 0.004 | 0.97 (0.94, 0.99) | 0.01 |
| Residual Model^**^ | 1.00 (ref) | 0.83 (0.60, 1.16) | 0.75 (0.53, 1.06) | 0.53 (0.36, 0.78) | 0.78 (0.54, 1.13) | 0.02 | 0.98 (0.96, 1.00) | 0.04 |
| Dietary patterns^††^ | 1.00 (ref) | 0.90 (0.65, 1.24) | 0.68 (0.48, 0.97) | 0.67 (0.46, 0.97) | 0.67 (0.44, 1.02) | 0.01 | 0.97 (0.95, 1.00) | 0.04 |

Abbreviations: CI: confidence interval, HR: Hazard ratio, PNNS-GS2: Programme National Nutrition Santé-guideline score 2, Q: quintile, T2D: type 2 diabetes

^*^ Values are mean differences (95% confidence intervals). 5 points correspond to 5% of the contribution of OF to the diet. Model are adjusted for age (time-scale), gender, familial history of diabetes, physical activity, occupation, marital status, education, monthly income per unit, smoking status, sPNNS-GS2, energy intake, alcohol consumption and body mass index.

^†^P for continuous independent variable

^‡^T2D cases occurring during the first year of follow-up were excluded from the sample (final n=33,162. 199 cases)

^§^Participants developing a cardiovascular disease during the follow-up or before were excluded from the sample (final n=32,468, 273 cases)

^||^Participants with hypertension or dyslipidemia at baseline were removed (final sample n=24,316, 85 cases)

^¶^Model was further adjusted for consumption of processed meat, whole grain and sweetened beverages

^#^Model was further adjusted for intake of saturated fatty acids, sugar, sodium and fiber

^**^Residual of the regression of organic food consumption on sPNNS-GS2 was considered as principal exposure

^††^Model with sPNNE-GS2 replaced by 4 dietary factors extracted by principal component analysis

**S3 Table: Mediation of the association between organic food consumption and risk of T2D by a healthy diet, NutriNet-Santé study, 2014-2019, N=33,256***

|  | **HR_Q2 vs Q1_** | **HR_Q3 vs Q1_** | **HR_Q4vs Q1_** | **HR_Q5vs Q1_** | **P for trend** |
| --- | --- | --- | --- | --- | --- |
| **Direct effect** | 0.89 (0.76, 1.05) | 0.68 (0.57, 0.81) | 0.66 (0.55, 0.79) | 0.72 (0.58, 0.88) | <0.0001 |
| **Indirect effect** | 0.97 (0.81, 1.17) | 0.94 (0.78, 1.13) | 0.90 (0.75, 1.09) | 0.81 (0.66, 0.99) | 0.03 |
| **Mediation**^†^ (%) | 18 | 12 | 15 | 32 |  |

Abbreviations: Q, Quintile; HR, hazard ratio.

^*^ The direct effect corresponds to the direct relation between organic food consumption and T2D, and the indirect effect corresponds to the relation mediated by sPNNS-GS2 (diet quality, in deciles) as described in the supplemental material.

Values are hazard ratio and 95% confidence interval adjusted for age (time-scale), gender, family history of diabetes, physical activity, occupation, marital status, education, monthly income per unit, smoking status, energy intake, alcohol consumption and body mass index

^†^Values correspond to the percentage of the total association mediated by sPNNS-GS2.

**S4 Table: Association between the proportion of organic food in the diet and risk of T2D, NutriNet-Santé study adjusted for the MEDI-LITE score, NutriNet-Santé study, 2014-2019, N=33,256***

| **model** | **Q1** | **Q2** | **Q3** | **Q4** | **Q5** | **P for trend^1^** | **5 points increment** | **P^2^** |
| --- | --- | --- | --- | --- | --- | --- | --- | --- |
| ***Total organic food*** |  |  |  |  |  |  |  |  |
| Person-years | 26,709 | 27,128 | 27,218 | 27,120 | 26,816 |  |  |  |
| Number of cases | 83 | 72 | 51 | 48 | 39 |  |  |  |
| Model 1^3^ | 1.00 (ref) | 0.93 (0.68, 1.28) | 0.66 (0.46, 0.93) | 0.65 (0.45, 0.93) | 0.55 (0.37, 0.81) | 0.0003 | 0.96 (0.93, 0.98) | 0.001 |
| Model 2^4^ | 1.00 (ref) | 0.87 (0.63, 1.20) | 0.66 (0.46, 0.93) | 0.62 (0.43, 0.90) | 0.55 (0.37, 0.82) | 0.0004 | 0.96 (0.94, 0.99) | 0.002 |
| ***Organic plant food*** |  |  |  |  |  |  |  |  |
| Person-years | 26,733 | 27,141 | 27,179 | 27,239 | 26,698 |  |  |  |
| Number of cases | 83 | 67 | 55 | 40 | 48 |  |  |  |
|  |  |  |  |  |  |  |  |  |
| Model 3^5^ | 1.00 (ref) | 0.88 (0.63, 1.21) | 0.74 (0.53, 1.05) | 0.55 (0.38, 0.81) | 0.76 (0.52, 1.10) | 0.01 | 0.97 (0.95, 1.00) | 0.03 |
| Model 4^6^ | 1.00 (ref) | 0.83 (0.60, 1.16) | 0.75 (0.53, 1.06) | 0.52 (0.36, 0.77) | 0.77 (0.53, 1.12) | 0.01 | 0.98 (0.95, 1.00) | 0.03 |
| ***Organic animal food*** |  |  |  |  |  |  |  |  |
| Person-years | 26,541 | 27,104 | 27,236 | 27,148 | 26,960 |  |  |  |
| Number of cases | 75 | 58 | 65 | 46 | 49 |  |  |  |
|  |  |  |  |  |  |  |  |  |
| Model 5^7^ | 1.00 (ref) | 0.81 (0.57, 1.14) | 0.93 (0.66, 1.30) | 0.68 (0.47, 0.99) | 0.84 (0.58, 1.22) | 0.19 | 0.99 (0.96, 1.01) | 0.27 |
| Model 6^8^ | 1.00 (ref) | 0.77 (0.54, 1.09) | 0.94 (0.67, 1.31) | 0.64 (0.44, 0.93) | 0.82 (0.56, 1.19) | 0.15 | 0.99 (0.96, 1.01) | 0.73 |

Abbreviation: Q: sex-specific quintile

^*^Values are Hazard ratio (95% confidence intervals), 5 points correspond to 5% of the contribution of OF to the diet

^1^P for trend modeling quintile as ordinal independent variable

^2^P for continuous independent variable

^3^Model 1 is adjusted for age (time-scale), gender, familial history of diabetes, physical activity, occupation, marital status, education, monthly income per unit, smoking status, MEDI-LITE, energy intake and alcohol consumption

^4^Model 2 is model 1 adjusted for body mass index

^5^Model 3 is model 1 adjusted for total plant food consumption

^6^Model 4 is model 3 adjusted for body mass index

^7^Model 5 is model 1 adjusted for total animal food consumption

^8^Model 6 is model 5 adjusted for body mass index

**Supplemental Material:**

A set of sensitivity analyses was conducted.

First, we reanalyzed the data after removing 1) early T2D cases, occurring during the first year of follow-up to eliminate potential reverse causality, 2) participants with cardiovascular diseases occurred before or during the follow-up, 3) participants with cardiometabolic risk factor at baseline (hypertension and/or dyslipidemia).

Second, several additional adjustments were tested: 1) nutrients intake, namely saturated fatty acids, sugar, sodium and fiber, 2) food groups associated with diabetes at a high level of evidence, namely processed meat, whole grain and sweet beverages, 3) sPNNS-GS2 was replaced by 4 factors extracted by principal component analysis: these factors were based on 33 food groups. A rotation was applied to simplify the construction of dietary patterns; the Cattel diagram claimed for the selection of 4 factors explaining 26% of the initial variability, 4) for comparability purpose, the main analysis were rerun by replacing the sPNNS-GS2 by the Medi-lite score (1). As previously published, the MEDI-LITE, ranging from 0 (least healthy) to 18 (healthiest), includes 9 components focusing on consumption of fruit, vegetables, whole grains, nuts and legumes, olive oil (positive points), dairy, red and processed meat (negative points), and alcohol (points according to consumption). Points are allocated according to a scoring system based on daily or weekly consumption.

Third, as the positive link between nutritional quality of the diet and the consumption of OF is well-known, besides using the common adjustment and stratification approaches, we conducted two additional analyses to improve disentangling the role of OF consumption from that of healthy food on the risk of T2D.

We modeled the residues of the regression of OF consumption on sPNNS-GS2 (reflecting the level of compliance with the French 2017 food guidelines).

Then, we quantified the mediating role of healthy diet (using the sPNNS-GS2 as mediator) in the association between OF consumption and the risk of T2D. For this purpose, the counterfactual-based mediation analysis proposed by Lange et al. (2) was used. It included the following steps:

1) A new dataset repeating each observation 5 times, including a new variable X*, corresponding to each of the 5 OF consumption quintiles. The new variable X* was equal to X (the observed OF consumption quintile) for one line per subject and different from X for the 4 other lines.

2) Two multivariable logistic regression models were then applied to the new dataset to estimate the association between OF consumption quintile (X/X*) and sPNNS-GS2 deciles (M), first using the original variable X, and then the new variable X*;

3) Weights were calculated using the predicted probabilities from the two logistic regressions, as follows:

$$\boldsymbol{W}_{\boldsymbol{i}}^{\boldsymbol{C}}\boldsymbol{=P(M=}\boldsymbol{M}_{\boldsymbol{i}}\boldsymbol{|X=}\boldsymbol{X}_{\boldsymbol{i}}^{\boldsymbol{*}}\boldsymbol{, C=}\boldsymbol{C}_{\boldsymbol{i}}\boldsymbol{)/P(M=}\boldsymbol{M}_{\boldsymbol{i}}\boldsymbol{|X=}\boldsymbol{X}_{\boldsymbol{i}}\boldsymbol{, C=}\boldsymbol{C}_{\boldsymbol{i}}\boldsymbol{)}$$

with *i:* the individual; *X:* the observed exposure; *X*:* the created exposure (indirect effect); *M:* the mediator; *C:* confounding factors.

4) Cox hazard proportional regression model adjusted for confounders was performed to estimate the association between OF consumption quintile (X and X*) and T2D. The HR associated with the X was interpreted as the direct effect and the HR associated with X* as the indirect effect.

5) Proportion (%) mediated by healthy diet was computed using the following formula:

$\left[ \mathrm{RR}^{\mathrm{NDE}}\left( RR^{\mathrm{NIE}}-1 \right) \right]/ [\mathrm{RR}^{\mathrm{NDE}} \times RR^{\mathrm{NIE}}-1) \times100$

where NDE is natural direct effect and NIE is natural indirect effect.
